# Individualised prediction of major bleeding in patients with atrial fibrillation treated with anticoagulation

**DOI:** 10.1371/journal.pone.0312294

**Published:** 2024-11-14

**Authors:** Anne Pernille Toft-Petersen, Christina J.-Y. Lee, Matthew Phelps, Brice Ozenne, Thomas Alexander Gerds, Christian Torp-Pedersen

**Affiliations:** 1 Department of Cardiology and Clinical Research, Nordsjællands Hospital, University of Copenhagen, Hillerød, Denmark; 2 The Danish Heart Foundation, Copenhagen, Denmark; 3 Department of Public Health, Section of Biostatistics, University of Copenhagen, Copenhagen, Denmark; 4 Neurobiology Research Unit and BrainDrugs, Copenhagen University Hospital Rigshospitalet, Copenhagen, Denmark; 5 Department of Biostatistics, University of Copenhagen, Copenhagen, Denmark; Qatar University, QATAR

## Abstract

**Background:**

Anticoagulation in atrial fibrillation (AF) increases the risk of major bleeding. No predictive model has hitherto provided estimates of the absolute risk for individual patients.

**Aim:**

To predict the individual 1-year risk of major bleeding in patients with AF taking anticoagulants and evaluate the importance of individual risk factors.

**Design:**

A nationwide register-based cohort study.

**Participants:**

Danish patients with first-time non-valvular AF who redeemed anticoagulants within 7 days after diagnosis.

**Method:**

The individual absolute risk of major bleeding was estimated from a logistic regression model (the Calculator of Absolute Bleeding Risk/CABS model) utilising the same risk factors as HAS-BLED, except allowing non-linear age effects, and allowing effect modification of all factors according to history of bleeding. The logistic regression was assessed in term of discrimination using the Area Under the ROC curve (AUC) and calibration.

**Results:**

Among 76,102 patients with AF redeeming anticoagulants, 2,406 suffered a major bleeding within 1 year. History of bleeding was the strongest predictor, and age significantly modified the risk. The CABS model superseded HAS-BLED score with regards to discrimination (AUC 0.646 vs 0.615, p<0.001) and calibrated well. A typical male patient was 70-years old without any risk factors and he had a 1-year bleeding risk of 1.4% (1.2; 1.6) while a typical female patient was 73-years old, had hypertension and a 1-year bleeding risk of 2.2% (1.9;2.6).

**Conclusion:**

We propose CABS as a tool for prediction of individual absolute risks of major bleeding in patients with AF taking anticoagulant. The predicted absolute risk can be used for patient counselling.

## Introduction

Bleeding is the most important complication to anticoagulant therapy in atrial fibrillation (AF), and fear of bleeding often prompts clinicians to withhold anticoagulation [1–4]. Bleeding, however, does also occur in patients with AF not treated with anticoagulants and the proportion of risk that can be attributed to anticoagulation per se is not known.

Before initiation of anticoagulation treatment individual bleeding risk assessments is recommended in both European and US guidelines. Both guidelines recommend that the resulting predicted bleeding risks are employed for flagging up modifiable risk factors while solely the risk of stroke determines whether anticoagulants should be initiated [5,6]. However, studies have shown that clinicians often withhold anticoagulants because of a subjective perception of a bleeding risk that outweighs the benefits of anticoagulation in a given patient [4].

Reported incidence of major bleeding in “real life” AF populations ranges from 3/100 person-years at risk (pyr) to 6/100 pyr in patients not on anticoagulants and from 1/100 pyr to 6/100 pyr in patients on anticoagulants [7–14]. While anticoagulants in themselves increase the risk of major bleeding in AF [15], the risk has also been shown to depend on patient specific factors.

Several clinically applicable and simple systems have been developed for assessment of the risk of bleeding with anticoagulant treatment in different settings [16], and the most commonly employed system is the HAS-BLED which is an additive score that encompasses clinical and paraclinical risk factors [17]. While identification of factors that puts an individual patient at increased risk of bleeding can guide management of said risk factors, neither of the commonly employed risk scoring systems (HAS-BLED, ATRIA [18], ORBIT [11], HEMORR_2_HAGES [19]) can provides guidance as to whether anticoagulation poses a disproportionate risk for an individual patient as they do provide relative categories of risk rather than predictions of individual, absolute risks. In the case of HAS-BLED, some attempts at calibration have been made and as it is per se not a model (with estimates of risk) but a score (with arbitrary points), the risks from the original derivation cohort have been utilised [20,21]. Similar HAS-BLED scores and thus predicted risks are therefore assigned to patients with different combinations of risk factors, which combinations in themselves associate with different risks [17].

We set out to build a new risk prediction model, the Calculator of Absolute Bleeding Risk (CABS) model, that would predict individual absolute risks of major bleeding in patients with AF taking anticoagulants. We benchmarked the CABS model against HAS-BLED, and in addition we used the predictions from the CABS model to examine the individual risk factors and their interplay.

The aims of the present study were thus 1) to quantify the absolute risk of major bleeding in real-life patients exposed to anticoagulants, and 2) to revisit the framework of the HAS-BLED score in order to improve our understanding of the individual risk factors.

## Material and methods

### Study population

The target population encompassed Danish anticoagulation-naïve first-time patients with AF without valvular disease who redeemed anticoagulants within 7 days after a first-time diagnosis of AF. The Civil Registration System [22] (CRS), the National Patient Registry (DNPR) [23] and the Registry of Medicinal Product Statistics (DRMPS) [24] (See S1 Table for definitions) were linked through the unique person identifier provided for all Danish residents. Admission and discharge dates for all hospital admissions and outpatient clinic visits from 1997 through 2018 where AF or atrial flutter was either the primary or a secondary diagnosis were extracted as well as dates of birth, sex, migration, and date of death. Surgical procedure codes for mitral or aortic valve surgery and discharge diagnoses of valvular disease and medical conditions were likewise extracted, and this information combined with information on redeemed prescriptions to determine if comorbidities were present at baseline. Major bleeding was defined as any of intracranial bleeding, bleeding in the spinal cord, gastrointestinal bleeding; bleeding in the joints, bleeding in the muscles (compartment syndrome), bleeding from the respiratory tract, pericardial bleeding, retroperitoneal bleeding, bleeding in eye and related structures, and anaemia from acute or chronic bleeding [25]. Anticoagulant treatment was identified from redeemed prescriptions for vitamin K antagonists and direct oral anticoagulants.

Patients with first-time AF admissions/outpatient visits from January 1, 1997 to December 31, 2018 were eligible for the study population at day 7 (the enrolment date) after discharge/outpatient visit (the AF date) provided that they were between 20 and 100 years old, had no valvular disease, had resided in Denmark for at least 5 years before the diagnosis of AF, and had not redeemed prescriptions for anticoagulants within the last 6 months before the AF date (i.e. were anticoagulation-naïve). Patients were excluded no matter whether anticoagulants should have been prescribed according to the guidelines at the time or not. Potentially eligible patients were excluded if stroke, major bleeding or death occurred within the first 7 days after the diagnosis of AF (the blanking interval). This exclusion ensured that events with uncertain order during an admission and potentially related to the clinical instability of patients at point of admission did not obfuscate the model of the one-year risk. Likewise, potentially eligible patients who did not redeem a prescription for anticoagulants in the blanking interval were excluded from the study. In the sensitivity analyses, the blanking interval was set to 60 days to assess whether delays in prescription or redemption ascribable to diagnostic uncertainty and outstanding assessments could have affected the results. Discontinuation of anticoagulants during follow-up was ignored and the risk estimated is therefore based on “intention to treat”.

Modified HAS-BLED scores (the sum of points obtained after assigning one point for each of hypertension, abnormal renal function, abnormal liver function, previous stroke, previous major bleeding, age above 65, and drug consumption or alcohol abuse) were calculated from baseline characteristics [26]. Labile INRs, which contribute a point in the HAS-BLED score, were not included, as patients were anti-coagulation naïve at enrolment.

Patients were followed up for a year after the enrolment date (the prediction time horizon) or until death, the first occurrence of major bleeding or date of censoring, December 31, 2019, whichever came first. Patients who emigrated without outcome before the prediction time horizon (n<15/year) were treated as no outcome. This introduces a neglectable negative bias into the risk estimates.

### Statistical methods

Patient characteristics at baseline were presented as medians with interquartile ranges (IQRs) or frequencies with percentages as appropriate. The baseline date for all analyses was set at 7 days after discharge, i.e., the enrolment date. The primary outcome was major bleeding within 1-year. The CABS model was pre-specified and built using logistic regression including the traditional HAS-BLED risk factors, age in years at enrolment date and calendar year as follows. Patient age and calendar date entered with non-linear effects via restricted cubic splines [27]. The risk factors hypertension, abnormal renal function, abnormal liver function, previous stroke drug consumption and alcohol abuse entered as binary variables with additive effects. All regression coefficients were modified by history of bleeding (interaction). Presented are patient individual 1-year risk predictions obtained by estimating the CABS model in data from all years (1997–2018). The model specification can be found in S2 Table.

To evaluate the predictive performance of our model in new patients, data was randomly split into training (63.2% of the data) and test (the remaining) datasets. The model was constructed using the training dataset. The predictive performance (area under the curve (AUC), calibration plot (= reliability diagram [28]), and Brier Score) was then estimated in patients in the test dataset. In addition to the analysis of prediction performance based on a random split of the data we also performed a calendar time split, where the CABS model was trained based on AF patients diagnosed before Dec 31, 2014, and the prediction performance estimated on AF patients diagnosed in the period between Jan 1, 2015 and Dec 31, 2018. The AUC quantifies the probability that the risk predicted for someone with bleeding is higher than the risk predicted for someone without bleeding. It thus measures the discriminative ability of the model with 1 as the highest value and 0.5 representing chance. A calibration plot shows observed (actual) against expected (predicted) risks in by the level of risk and presents visually how accurate the model predicts at different levels of risk. The calibration of a model is crucial to interpretation as even highly discriminative models can grossly overestimate risk and as calibration may highlight that a model misrepresents risk in certain risk groups. The Brier score is a compound measure of discrimination and calibration ranging from 0 to 1, where a score of 0 represents perfect accuracy.

To ease the interpretation of the performance of our model, a logistic regression model which included the HAS-BLED score (point score 1–8) as a categorical variable was used as a benchmark model.

The level of statistical significance was set at 5%. SAS version 9.4 (SAS Institute, Cary, NC), and R version 4.2.1 [29] were employed.

Danish studies that are conducted for statistics and scientific research purposes do not require ethical approval or informed consent [30]. This study was conducted in compliance with the Danish Data Protection Act and the General Data Protection Regulation and approved by the data responsible unit in the Capital Region of Denmark. Data were assessed repeatedly but no later than June 1, 2024. Authors did not have access to information that could identify individual participants during or after data collection.

## Results

### Population

**Fig 1:**
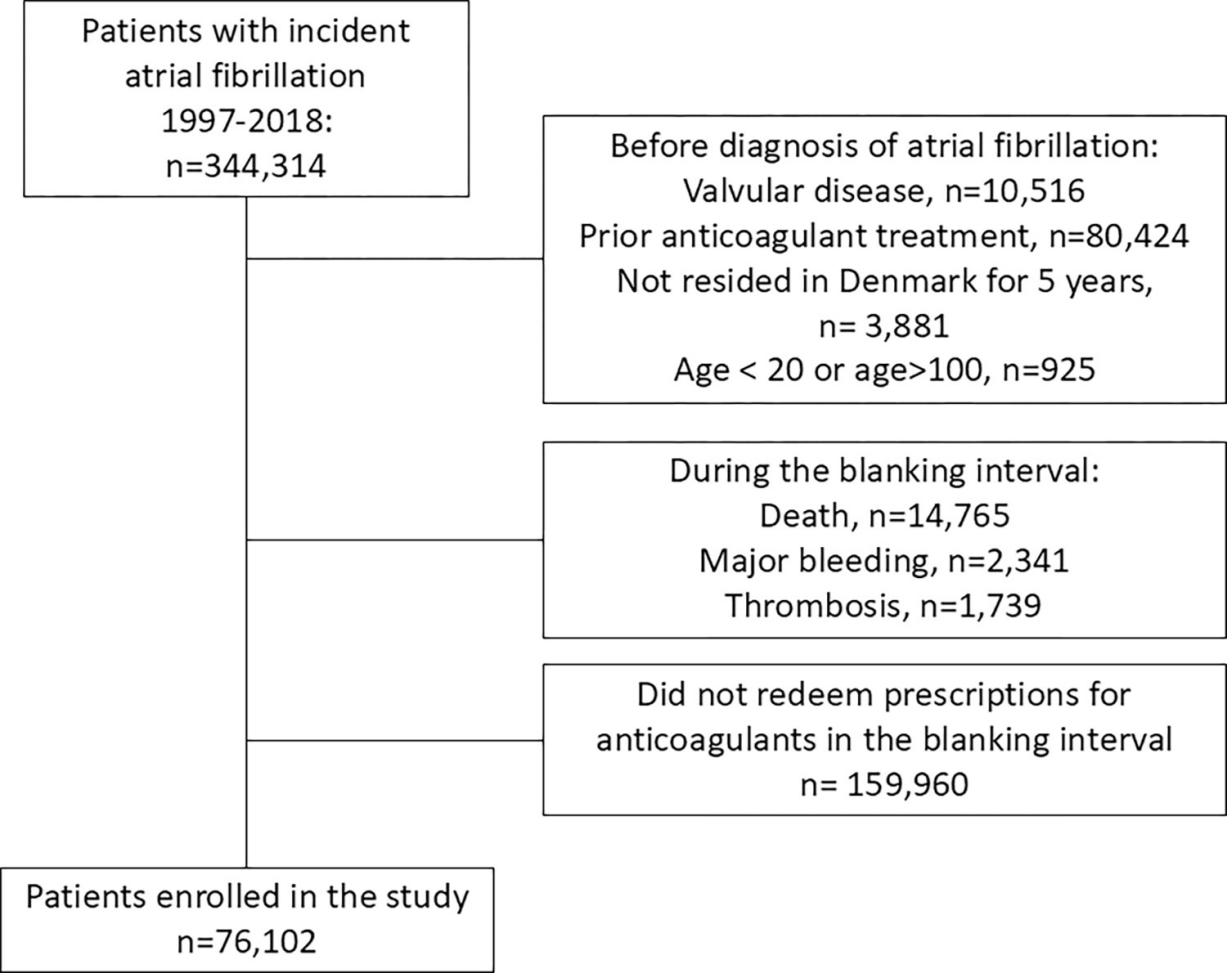
The study population.

We identified 344,314 Danish patients with incident AF in the 20-year period of enrolment. Of these, 76,102 patients were eligible for study entry at day 7 after initial admission, the enrolment date (Fig 1).

The case-mix of the included patients according to their HAS-BLED scores is shown in Table 1. A minority of the patients were women 34,407 (45.2%) and the median age was 74.2 years [IQR 66.6–81.1]. Vitamin K antagonists were the most frequently redeemed anticoagulants. Only 5.5% of patients were assigned zero HAS-BLED points whereas 53.4% were assigned one or two points. HAS-BLED points were most frequently assigned based on age > 65 (79.1%), hypertension (65.0%), and drug consumption (52.1%).

**Table 1:** The case-mix of included patients stratified by HAS-BLED scores at baseline.

Variable	Level	HAS-BLED = 0 (n = 4,150)	HAS-BLED = 1–2 (n = 40,620)	HAS-BLED >2 (n = 31,332)	Total (n = 76,102)
Age	20–49	971 (23.4)	1,165 (2.9)	106 (0.3)	2,242 (2.9)
	50–64	3,179 (76.6)	9,278 (22.8)	1,225 (3.9)	13,682 (18.0)
	65–74	0 (0.0)	13,264 (32.7)	11,129 (35.5)	24,393 (32.1)
	75–100	0 (0.0)	16,913 (41.6)	18,872 (60.2)	35,785 (47.0)
Female	yes	1,003 (24.2)	18,336 (45.1)	15,068 (48.1)	34,407 (45.2)
Congestive heart failure	yes	756 (18.2)	15,617 (38.4)	15,291 (48.8)	31,664 (41.6)
Hypertension	yes	0 (0.0)	20,684 (50.9)	28,796 (91.9)	49,480 (65.0)
Diabetes	yes	182 (4.4)	4,028 (9.9)	6,230 (19.9)	10,440 (13.7)
Previous stroke	yes	0 (0.0)	1,817 (4.5)	9,126 (29.1)	10,943 (14.4)
Vascular disease	yes	48 (1.2)	2,504 (6.2)	5,630 (18.0)	8,182 (10.8)
Abnormal renal function	yes	0 (0.0)	267 (0.7)	2,526 (8.1)	2,793 (3.7)
Abnormal liver function	yes	0 (0.0)	123 (0.3)	660 (2.1)	783 (1.0)
Previous major bleeding	yes	0 (0.0)	757 (1.9)	4,132 (13.2)	4,889 (6.4)
Drug consumption	yes	0 (0.0)	11,457 (28.2)	28,192 (90.0)	39,649 (52.1)
Alcohol abuse	yes	0 (0.0)	505 (1.2)	1,340 (4.3)	1,845 (2.4)
Type of anticoagulant	Vitamin K antagonist	2,664 (64.2)	24,113 (59.4)	18,866 (60.2)	45,643 (60.0)
	Direct oral anticoagulant	1,481 (35.7)	16,445 (40.5)	12,422 (39.6)	30,348 (39.9)
	Indeterminable	5 (0.1)	62 (0.2)	44 (0.1)	111 (0.1)
CHA_2_DS_2_VASc score	0	2,489 (60.0)	924 (2.3)	6 (0.0)	3,419 (4.5)
	1	1,350 (32.5)	5,705 (14.0)	169 (0.5)	7,224 (9.5)
	> = 2	311 (7.5)	33,991 (83.7)	31,157 (99.4)	65,459 (86.0)

### Outcomes

One year after enrolment, 90.7% of included patients were still alive. The incidence rate of major bleeding within the first year was 34.0/1,000 pyr (Table 2). Most of those major bleedings were gastrointestinal (17.8/1,000pyr) and intracranial bleedings only amounted to 6,4/1,000pyr. The incidences of major bleeding were higher with HAS-BLED 1–2 or >2 than with HAS-BLED 0 as were the incidences of intracranial and gastrointestinal bleeding. The incidence of stroke was higher than the incidence of major bleeding with zero HAS-BLED points, but major bleeding dominated when HAS-BLED was 1 or 2.

**Table 2:** The incidence rates per 1,000 person-years of bleeding, stroke, and death according to HAS-BLED scores.

	HAS-BLED = 0	HAS-BLED = 1–2	HAS-BLED = >2	Total
Major bleeding	5.85	25.26	49.97	34.00
Gastrointestinal bleeding	2.92	12.84	26.54	17.78
Intracranial bleeding	0.73	5.08	9.01	6.40
Stroke	8.55	23.02	58.71	36.43
Death	15.55	77.09	139.08	98.51

The risks predicted by the CABS model ranged between 0.43% and 14.2% with a median of 3.9% for those with major bleeding and between 0.21% and 19.3% with a median of 2.77% for those without major bleeding.

**Fig 2:**
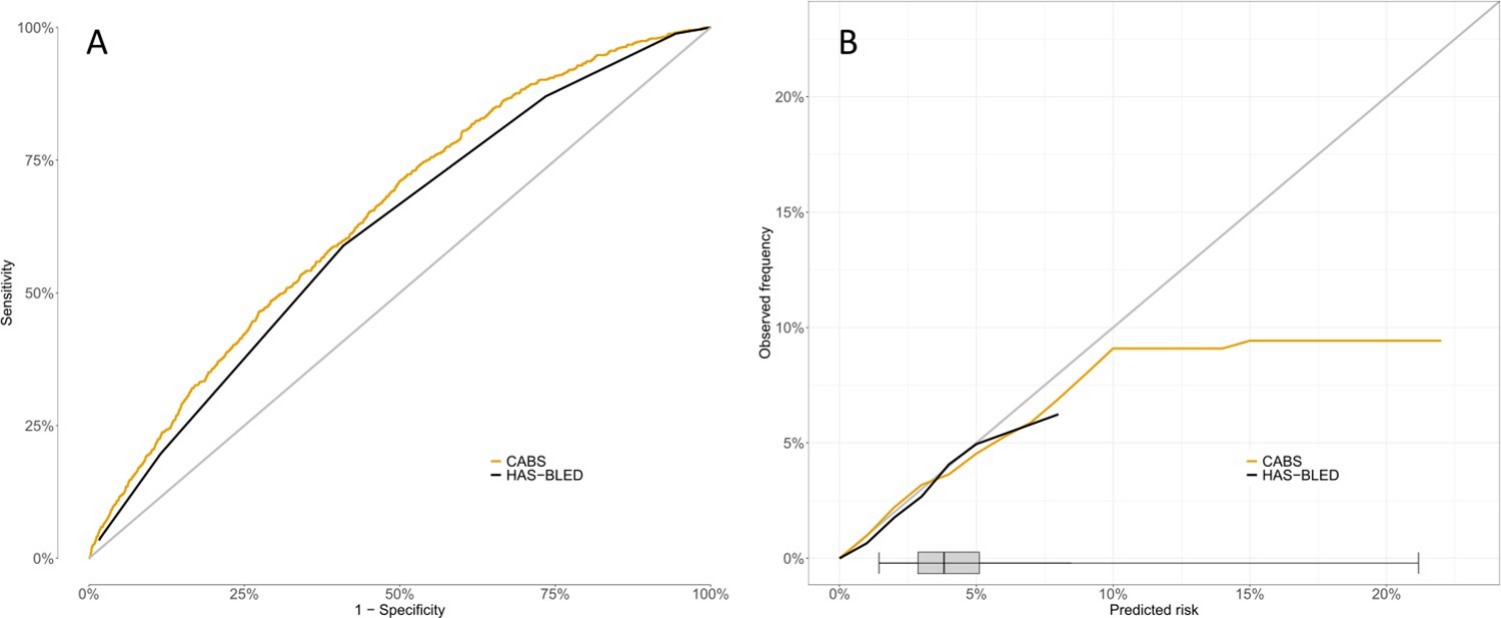
The performance of the CABS-model. A: Discrimination of the CABS model vs the HAS-BLED score, B: The calibration of the CABS model vs the HAS-BLED across bleeding risk by predicted risk increments. Along the x-axis is shown the distribution of predicted risks.

The discrimination of the CABS model was (AUC = 0.646, CI = [0.629;0.664]) and superseded that of the HAS-BLED score (AUC = 0.615, CI = [0.597;0.632], p = 0.04) (Fig 2A). Brier scores were similar in the two models (2.97% for CABS vs 2.98% for HAS-BLED, p>0.05). The CABS model showed good calibration in low- and high-risk patients alike but trailed off above a predicted risk of 10% (Fig 2B).

The calendar time split analysis also showed an AUC of 0.654 (CI = [0.636;0.671]) for CABS compared to AUC for HAS-BLED of 0.613 (CI = [0.595;0.630]). The difference in AUC was significant 4.1% (CI = [2.9;5.4], p-value<0.001).

**Fig 3:**
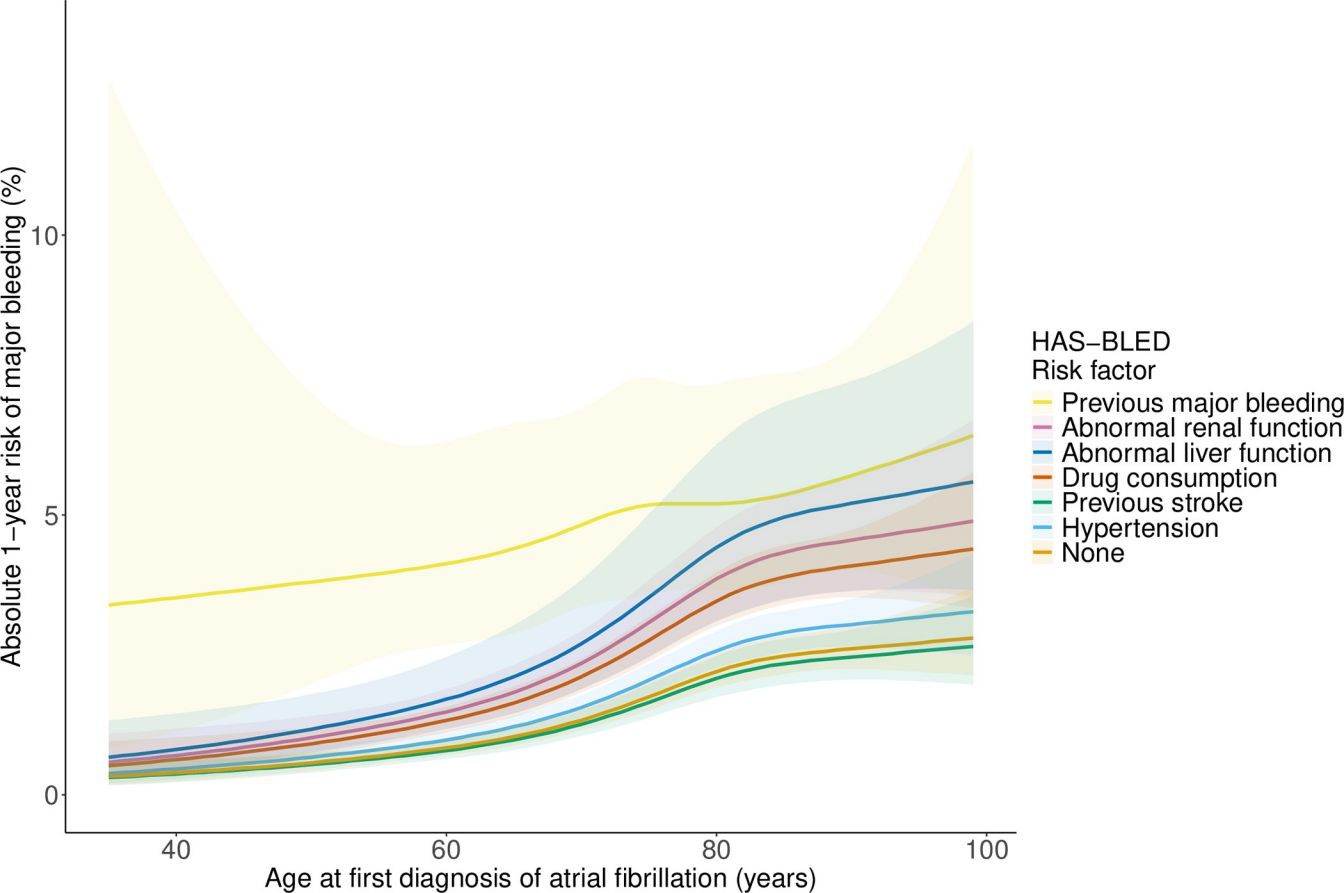
The one-year predicted risk of major bleeding with no risk factor (black curve) or any one risk factor by age.

Breaking down the HAS-BLED score to individual risk factors revealed significantly different associations between the individual comorbidities across age and the absolute risk of major bleeding predicted from the CABS model (Fig 3). Predicted risk increased monotonously with age. The risk associated with previous major bleeding was more than twice as large as the risk associated with hypertension and previous stroke. Intermediate strengths of association were seen for chronic kidney disease, drugs, and liver disease. The associations were essentially unaffected by sex. Among young patients, only previous major bleeding was associated with a substantially elevated risk.

**Fig 4:**
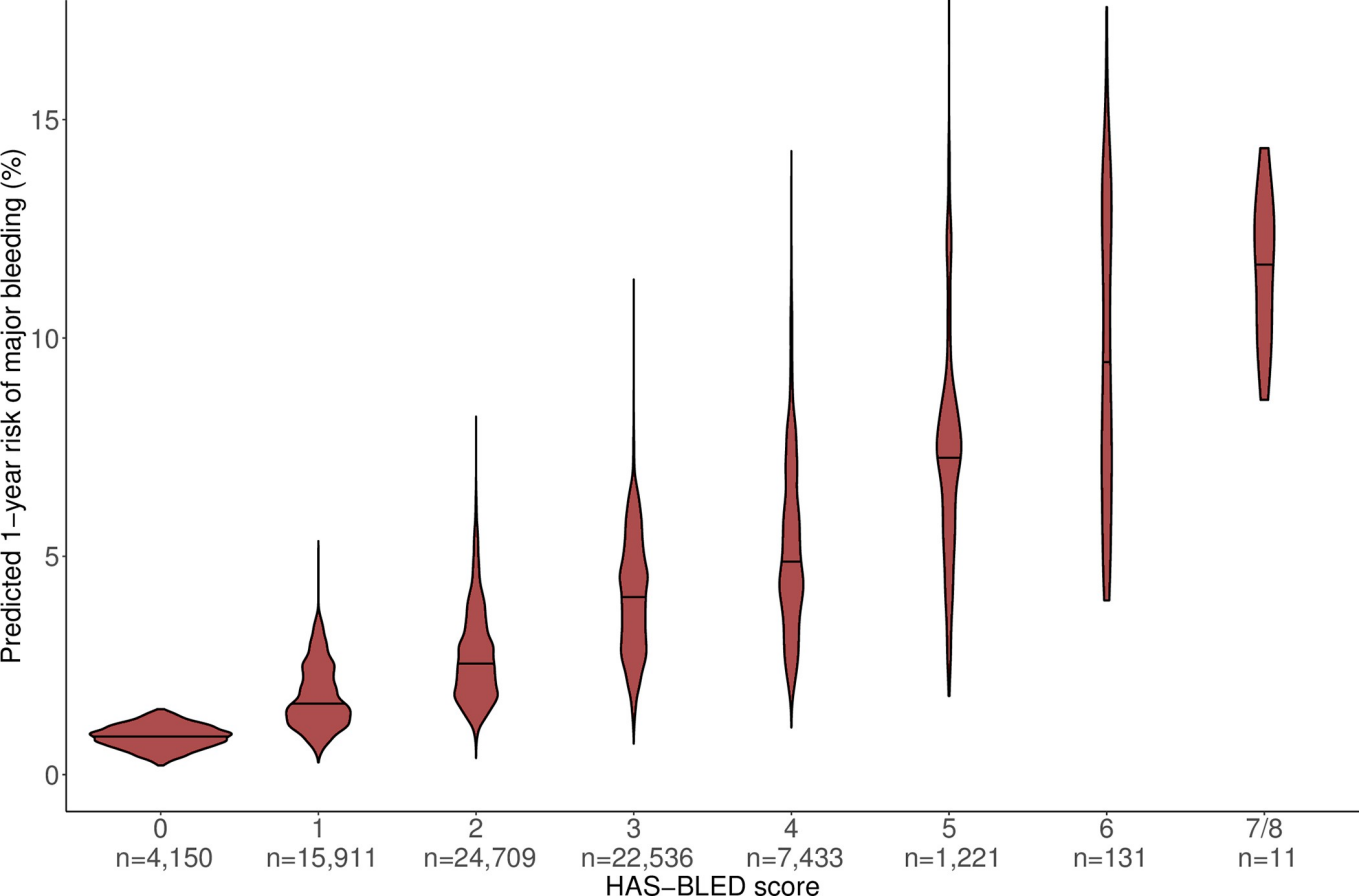
The absolute one-year risk of major bleeding predicted from CABS by HAS-BLED score. The column width shows the within-column distribution of predicted risk. The number of patients assigned a given HAS-BLED score is indicated at the x-axis. No patients had a HAS-BLED score = 8.

Fig 4 shows the absolute one-year risk of major bleeding predicted from the CABS model by HAS-BLED score. The median predicted risk increases monotonously with the HAS-BLED score up to 6, above which point data is scarce. There are large variabilities in the predicted absolute risks with extensively overlapping distributions. The predicted risk distributions within the individual HAS-BLED scores have distinct patterns, reflecting different weights of the risk factors.

In a sensitivity analysis where the blanking interval was set to 60 days, 58.1% of otherwise eligible patients were excluded because they did not redeem anticoagulants. When patients were followed from day 60 instead of day 7 after initial AF contact, the AUC for the CABS model was 0.632 and for the HAS-BLED score 0.613 (p<0.01) (See S1 Fig).

## Discussion

In this study of first-time patients with AF, who commenced anticoagulation immediately after diagnosis, we found that the majority had one or more established risk factors for bleeding, and that major bleeding, with an incidence of 43.3/1000pyr, was fairly frequent though not as frequent as stroke. We built a complex, flexible model for prediction of major bleeding within a year from the same risk factors as utilised in HAS-BLED but with higher discriminative power—the CABS model. We compared this new CABS model to the HAS-BLED score and found that the median CABS predictions did increase monotonously with the HAS-BLED scores but that the individual risk predictions with any HAS-BLED score was too uncertain to be clinically meaningful.

In contrast to existing bleeding risk prediction scores/models, we have developed and trained CABS within a big real-world population, comparable with regards to distributions of age and sex to other populations in countries with public health care [31,32]. Interestingly, our population presents a comparatively high crude mortality rate, that most likely reflect that the clinical adherence to anticoagulant treatment is relatively high in Denmark [33]. In line with our findings, external validation of HAS-BLED as well as the other bleeding risk scores has most often yielded only moderate discrimination [20].

As the HAS-BLED score was originally developed from a small cohort (n = 3,978) with few bleeding events (1.5%) and as ease of use was prioritized, the same weight was assigned to all risk factors [17]. We suggest that this weighting as well as misspecification might be limiting factors in prediction as we find absolute risks of different magnitudes with individual risk factors. Tying in with this, the HAS-BLED score was outperformed by the ATRIA score for prediction of bleeding among patients not on anticoagulants [34]. The ATRIA score assigns only one point for previous bleeding, in contrast to the three points assigned for anaemia and severe renal disease, does not include previous stroke, and weights age by two points. Assuming that anaemia is a proxy for previous bleeding or for renal dysfunction, it seems probable that, by assigning three points in the ATRIA score, the importance of these risk factors is more appropriately captured. With a prevalence of 9%, previous major bleeding was a moderately frequent risk factor in our population and to add further complexity to the question of weighting, previous major bleeding behaved markedly different from the other HAS-BLED risk factors. This agrees with the inability of the CHA_2_DS_2_-VASc score (which shares some risk factors with HAS-BLED but not previous bleeding) to predict new bleedings in patients with previous intracranial haemorrhages, suggesting a subgroup of patients for whom prediction would require an entirely different framework [35].

The appropriacy of the use of the HAS-BLED score or indeed any bleeding score for decision-making has long been questioned and it has been suggested that the scores should be used for flagging up modifiable risk factors for intervention rather than as guidance on anticoagulation [36]. While several of the risk factors are indeed modifiable (hypertension, alcohol consumption, use of drugs, and, to some extent, renal and liver function) a number of patients will remain at increased risk of bleeding. While most patients will still overall benefit from anticoagulants, a minority may have disproportionately high bleeding risks which could warrant withholding of anticoagulants. Different studies have previously employed the bleeding risk scores to estimate absolute risks. The incidences of bleeding with different scores in the HAS-BLED derivation cohort [17] were used as predicted risks but did not consistently calibrate [11,20,21], and as only three risk levels (low, 1.13/100pyr; moderate, 1.33/100pyr; and high, 4.94/100pyr) were identified, the patients with risks of bleeding comparable to the risk of stroke were missed.

To our knowledge, Barnett-Griness et al. have published the only model for individual absolute risk prediction in an AF-population administered NOAC [37]. The model discriminated better than HAS-BLED, ORBIT, ATRIA and CHA_2_DS_2_-VASc and calibrated well across the range of risk. The approach was different to ours in that risk factors not generally available from public health registers (ie known fall risk) were included and in that interaction between risk factors was not allowed for. Age did not meet the cut for inclusion in the model, probably because the population was relatively old (mean age 78.7 years), and in contrast to HAS-BLED, risk factors were assigned different weights. It would be interesting to validate this model in our population and compare it to CABS, but we cannot provide information on the risk factors included.

Direct oral anticoagulants were introduced during our study period and have, since their introduction, become increasingly popular [38]. Most trials have found direct oral anticoagulants superior with respect to stroke prevention and all-cause mortality as well as bleeding risk [39]. Our study was not designed to allow a validation in subsets of patients administered anticoagulation.

The individualised risk prediction provided by the CABS model improved discrimination across the span of risk with good calibration in the densely populated risk sets and can therefore provide more clinically useful information for patient counselling and risk factor management. Nowadays, with the ubiquitous accessibility of computers, ease of calculation is of dwindling importance and as the advantages of absolute risk predictions on the individual level are being increasingly appreciated among clinicians, we suggest that higher complexity will not deter clinicians from employing a more accurate model.

### Strengths

Our study has a number of strengths. Firstly, the risk of information bias is small with the single-tiered Danish health care system where diagnoses and prescriptions are linked to reimbursement of the health care provider and the patient, respectively.

Secondly, we provide absolute risk estimates which, compared to relative risks, are more directly useful to clinicians [40]. Bearing in mind that selection of patients for anticoagulant treatment cannot be ruled out, absolute risks of bleeding and stroke can be compared head-to-head *within the group of patients not on anticoagulants* and unwarranted withholding or commencing of treatment identified.

### Limitations

There are noteworthy limitations to our study.

The positive predictive values of diagnoses in the Danish National Patient Registry are generally high [41] but the negative predictive values are unknown as are the negative predictive values of the definitions of diabetes and hypertension employed. The resulting misclassification, which is likely differential, i.e., high-risk patients are erroneously assigned low-risk status, would most likely lead to an overestimation of the true bleeding risk associated with these risk factors.

As there is no validated algorithm to decide if patients who died without being admitted to hospital died from bleeding, some events of bleeding were inevitably missed.

Importantly, the absolute risk of bleeding in the presence of even a known set of risk factors in any given AF patient in the absence of anticoagulation cannot be directly inferred from our data. Our cohort consists of patients a priori deemed eligible for anticoagulants and both this and other studies show that anticoagulation is more often withheld when risk factors are present [42]. Likewise, along with objective criteria, physicians are known to incorporate numerous subjective patient factors in the decision on anticoagulation [43] and as these cannot be picked up from our registers, selection on these parameters may bias our analysis. If indeed these objective and subjective factors predict bleeding, the bleeding risk predicted from our cohort will tend to be biased and lower than the true risk.

In our main analysis, we only included patients who redeemed prescriptions for anticoagulation within 7 days after a first-time diagnosis of AF and in the first sensitivity analysis within the first 60 days. A considerable proportion, 18.9%, of patients excluded from our cohort due to not initiating anticoagulants in the blanking interval did, however, redeem prescriptions for anticoagulation within the following year. The extent to which the CABS model can be generalised to those late initiators is unknown.

## Conclusion

The CABS model, built on the same risk factors as HAS-BLED, predicts the absolute risk of major bleeding in patients with AF taking anticoagulants but has improved discrimination and calibrates well. Future external validation is needed to show that CABS can be used for individual patient counselling with the caveat that bleeding is a less frequent and often less severe adverse outcome compared to stroke.

## Supporting information

S1 Table Codes for treatments, diagnoses, and procedures. (PDF)

S2 Table Model specification for CABS. (PDF)

S3 Table The case-mix of included patients stratified by HAS-BLED scores at baseline. Blanking interval set to 60 days.
(PDF)

S4 Table The incidence rates per 1000 person-years of bleeding, stroke, and death according to HAS-BLED scores. Blanking interval set to 60 days.
(PDF)

S1 Fig The performance of the CABS-model in patients who had redeemed anticoagulants within 60 days after a diagnosis of atrial fibrillation. A: Discrimination of the CABS model vs the HAS-BLED score, B: The calibration of the CABS model vs the HAS-BLED across bleeding risk by predicted risk increments. Along the x-axis is shown the distribution of predicted risks.
(TIF)

S2 Fig The one-year predicted risk of major bleeding ascribable to any one risk factor by age in patients who had redeemed anticoagulants within 60 days after a diagnosis of atrial fibrillation. (TIF)

S3 Fig The absolute one-year risk of major bleeding predicted from CABS by HAS-BLED score in patients who had redeemed anticoagulants within 60 days after a diagnosis of AF. The column width shows the within-column distribution of predicted risk and the number of patients assigned a given HAS-BLED score is indicated at the x-axis.
(TIF)
