# Supplementary material for: Individualised prediction of major bleeding in patients with atrial fibrillation treated with anticoagulation
Source: PLoS One. 2024 Nov 14;19(11):e0312294. doi: 10.1371/journal.pone.0312294 (PMC11563370; doi:10.1371/journal.pone.0312294)
Supplement: S1 Table — (PDF) [file pone.0312294.s001.pdf]

ATC = Anatomical Therapeutic Chemical Classification System

ICD8 = The International Classification of Diseases system, 8th revision

ICD10 = The International Classification of Diseases system, 10th revision

NOMESCO = The Nordic Medical Statistics Committees Classification of Surgical Procedures

**Atrial fibrillation or flutter**	**ICD8:** 42793, 42794<br>**ICD10:** DI48
**Valvular atrial fibrillation:**<br>*(Rheumatic affection of the valves, Artificial valve, valvular operation)*	**ICD8:** 39400, 39401, 39402, 39408, 39409, 39500, 39501, 39502, 39508, 39509, 39600, 39601, 39602, 39603, 39604, 39608, 39609, 99751<br>**ICD10:** DI05-06, DI080A, DI081A, DI082A, DI083A, DZ952, DZ954<br>**NCSP:** KFKD, KFKH, KFMD, KFMH, KFGE, KFJF
**Anticoagulants**	
**Vitamin K antagonist**	**ATC:** B01AA
**Direct oral anticoagulant**<br>(Rivaroxaban, Apixaban, Dabigatran)	**ATC:** B01AF01, B01AF02,<br>B01AE07
**Major bleeding**	
**Intracranial bleeding (ICH)**	**ICD10:** DI60-DI62, DS064-066,
**Gastrointestinal bleeding (GI)**	**ICD10:** DI850, DK250, DK252, DK254, DK256, DK260, DK262, DK264, DK266, DK270, DK272, DK274, DK276, DK280, DK282, DK284, DK286, DK290, DK625, DK661, DK920, DK921, DK922, DK228F, DK298A, DK638B, DK638C, DK838F, DK868G, DI864A
Respiratory tract bleeding	**ICD10:** DR04, DJ942
Ocular bleeding	**ICD10:** DH31, DH356, DH431, DH450, DH052A

Retroperitoneal bleeding	**ICD10:** DS368D
Intraspinal bleeding	**ICD10:** DG951A
Pericardial bleeding	**ICD10:** DI312
Articular bleeding	**ICD10:DM250**
Muscular bleeding (compartment syndrome)	**ICD10:DT796**
Anaemia	**ICD10:** DD62, DD500
**Hypertension** *(diagnosis and/or defining treatment)*	**ICD8:** 40009, 40019, 40029, 40039, 40099, 40199, 40299, 40399, 40499<br>**ICD10:** DI10-15<br>**ATC:**<br>Antiadrenergic: C02A, C02B, C02C<br>Diuretics (DIU): C02DA, C03A, C03B, C03D, C03E, C03X<br>Vaso: C02DB, C02DD, C02DG<br>Beta blockers (BB): C07A<br>Calcium channel blockers (CCB): C08<br>Renin Angiotensin System inhibitors (RAS):<br>C09AA, C09XA02, C09CA<br><br>BB + DIU: C07B, C07C, C07D, C07F<br>CCB + DIU: C08G<br>RAS + DIU: C09BA, C09DA, C09XA52<br>Other + DIU: C02L<br>RAS + CCB: C09BB, C09DB
**Abnormal renal or hepatic function**	
Chronic kidney disease (CKD)	**ICD8:** 582, 583,24902, 25002, 58499, 59009, 59320, 75310, 75311, 75319, 79299, 40399, 40499, 44609, 44629

	**ICD10:** DN02, DN03, DN04, DN05, DN06, DN07, DN08, DN11, DN12, DN14, DN16, DN18, DN19, DN25, DN26, DN158, DN159, DQ612, DQ613, DQ615, DQ619, DE102, DE112, DE142, DI120, DM300, DN313, DM319, DM321
Liver disease	**ICD8:** 15509, 15519, 15589, 45600, 45601, 57109, 57110, 57111, 57119, 57190, 57191, 57192, 57193, 57194, 57199, 57200, 57201, 57209, 57300, 57301, 57302, 57303, 57304, 57305, 57309<br>**ICD10:** DB18, DK71, DK72, DK73, DK74, DK75, DK76, DC22, DK70, DK77, DB942, DT864, DZ944, DD684, DI982, DQ618A
**Stroke** (includes Transient Cerebral Ischaemia and arterial embolism and thrombosis)	**ICD8:** 43309, 43399, 43409, 43499, 43600, 43601, 43609, 43690, 43699, 43700, 43701, 43708, 43709, 43790, 43791, 43798, 43799, 43809, 43899, 43509, 43599, 44400, 44408, 44409, 44419, 44420, 44421, 44428, 44429, 44439, 44440, 44441, 44442, 44443, 44444, 44448, 44449, 44490, 44499<br>**ICD10:** DI63, DI64, DG458, DG459, DI74
**Drugs**	
NSAIDs	**ATC:** M01A (excl M01AX05)
Antiplatelets	**ATC:** B01AC06, N02BA01, B01AC04, B01AC22, B01AC24, B01AC07
Alcohol	**ICD8:** 979, 980, 291, 303,57109, 57110, 57710, E8609<br>**ICD10:** DF10, DK70, DE52, DT51, DE244, DG312, DG621, DG721, DI426, DK292, DK852, DK860, DO354, DR780, DZ502, DZ714, DZ721, DL278A, DT500A, DZ721A, DZ721B
**Heart failure** *(Diagnosis and/or defining treatment)*	**ICD8:**425, 4270, 4271<br>**ICD10:** DI110, DI42, DI50, DJ819<br>**ATC:** C03C, C03EB

**Diabetes Mellitus**, type I and II *(Diagnosis and/or defining treatment)*	**ICD8:** 250<br>**ICD10:** DE10-14<br>**ATC:** A10
**Vascular disease**	**ICD8:** 44009, 44019, 44020, 44021, 44028, 44029, 44030, 44039, 44099, 410, 41109, 41199, 41209, 41299, 41309, 41399, 41409, 41499<br>**ICD10:** DI70, DI21-25
