# Supplementary material for: Individualised prediction of major bleeding in patients with atrial fibrillation treated with anticoagulation
Source: PLoS One. 2024 Nov 14;19(11):e0312294. doi: 10.1371/journal.pone.0312294 (PMC11563370; doi:10.1371/journal.pone.0312294)
Supplement: S2 Table — (PDF) [file pone.0312294.s002.pdf]

Factor	Odds ratio	95% CI
(Intercept)	0	[-8.29;-5.41]
bleeding	29.52	[-0.32;7.09]
stroke	0.94	[-0.18;0.06]
ckd	1.78	[0.40;0.76]
liver	2.05	[0.38;1.06]
rcs(age)age	1.04	[0.01;0.06]
rcs(age)age'	1.03	[-0.05;0.11]
rcs(age)age''	0.89	[-0.64;0.41]
rcs(age)age'''	0.92	[-1.06;0.88]
hypertension	1.17	[0.06;0.26]
drugs	1.59	[0.37;0.56]
rcs(year2000)year2000	1.02	[-0.02;0.06]
rcs(year2000)year2000'	1	[-0.12;0.12]
rcs(year2000)year2000''	0.73	[-0.83;0.21]
rcs(year2000)year2000'''	4.65	[-0.14;3.21]
bleeding:stroke	1.14	[-0.18;0.43]
bleeding:ckd	1.05	[-0.33;0.44]
bleeding:liver	0.56	[-1.32;0.17]
bleeding:rcs(age)age	0.97	[-0.09;0.03]
bleeding:rcs(age)age'	1	[-0.21;0.21]
bleeding:rcs(age)age''	0.88	[-1.57;1.32]
bleeding:rcs(age)age'''	1.77	[-2.11;3.25]
bleeding:hypertension	0.88	[-0.42;0.17]
bleeding:drugs	0.78	[-0.52;0.01]
bleeding:rcs(year2000)year2000	1	[-0.12;0.12]
bleeding:rcs(year2000)year2000'	0.89	[-0.48;0.26]

bleeding:rcs(year2000)year2000''	2.27	[-0.74;2.38]
bleeding:rcs(year2000)year2000'''	0.02	[-8.78;0.78]
