# Supplementary material for: Individualised prediction of major bleeding in patients with atrial fibrillation treated with anticoagulation
Source: PLoS One. 2024 Nov 14;19(11):e0312294. doi: 10.1371/journal.pone.0312294 (PMC11563370; doi:10.1371/journal.pone.0312294)
Supplement: S3 Table — Blanking interval set to 60 days. (PDF) [file pone.0312294.s003.pdf]

Variable	Level	HAS-BLED = 0 (n= 4,244)	HAS-BLED = 1-2 (n=46,855)	HAS-BLED >2 (n=40,228)	Total (n=91,327)
Age	20-49	1,050 (24.7)	1,489 (3.2)	149 (0.4)	2,688 (2.9)
	50-64	3,194 (75.3)	11,559 (24.7)	1,685 (4.2)	16,438 (18.0)
	65-74	0 (0.0)	14,808 (31.6)	14,349 (35.7)	29,157 (31.9)
	75-100	0 (0.0)	18,999 (40.5)	24,045 (59.8)	43,044 (47.1)
Female	yes	1,019 (24.0)	20,856 (44.5)	19,234 (47.8)	41,109 (45.0)
Congestive heart failure	yes	719 (16.9)	19,093 (40.7)	20,882 (51.9)	40,694 (44.6)
Hypertension	yes	0 (0.0)	25,034 (53.4)	37,048 (92.1)	62,082 (68.0)
Diabetes	yes	180 (4.2)	4,756 (10.2)	8,050 (20.0)	12,986 (14.2)
Previous stroke	yes	0 (0.0)	2,168 (4.6)	12,097 (30.1)	14,265 (15.6)
Vascular disease	yes	47 (1.1)	2,923 (6.2)	7,208 (17.9)	10,178 (11.1)
Abnormal renal function	yes	0 (0.0)	354 (0.8)	3,423 (8.5)	3,777 (4.1)
Abnormal liver function	yes	0 (0.0)	167 (0.4)	871 (2.2)	1,038 (1.1)
Previous major bleeding	yes	0 (0.0)	844 (1.8)	5,171 (12.9)	6,015 (6.6)

Variable	Level	HAS-BLED = 0 (n= 4,244)	HAS-BLED = 1-2 (n=46,855)	HAS-BLED >2 (n=40,228)	Total (n=91,327)
Drug consumption	yes	0 (0.0)	13,977 (29.8)	36,261 (90.1)	50,238 (55.0)
Alcohol abuse	yes	0 (0.0)	638 (1.4)	1,775 (4.4)	2,413 (2.6)
Type of anticoagulant	Vitamin K antagonist	2,794 (65.8)	28,258 (60.3)	24,820 (61.7)	55,872 (61.2)
	Direct oral anticoagulant	1,401 (33.0)	17,997 (38.4)	14,828 (36.9)	34,226 (37.5)
	Indeterminable	49 (1.2)	600 (1.3)	580 (1.4)	1,229 (1.3)
CHA2DS2VASc score	0	2,599 (61.2)	1,160 (2.5)	6 (0.0)	3,765 (4.1)
	1	1,338 (31.5)	6,594 (14.1)	214 (0.5)	8,146 (8.9)
	>=2	307 (7.2)	39,101 (83.5)	40,008 (99.5)	79,416 (87.0)
