# Supplementary material for: Individualised prediction of major bleeding in patients with atrial fibrillation treated with anticoagulation
Source: PLoS One. 2024 Nov 14;19(11):e0312294. doi: 10.1371/journal.pone.0312294 (PMC11563370; doi:10.1371/journal.pone.0312294)
Supplement: S4 Table — Blanking interval set to 60 days. (PDF) [file pone.0312294.s004.pdf]

	HAS-BLED = 0	HAS-BLED = 1-2	HAS-BLED = >2	Total
Major bleeding	5.25	22.33	41.75	29.86
Gastrointestinal bleeding	2.38	10.93	21.49	15.08
Intracranial bleeding	1.19	4.77	8.49	6.20
Stroke	6.21	19.69	52.59	33.19
Death	14.01	69.23	122.33	89.53
