# Supplementary figures and images for: Individualised prediction of major bleeding in patients with atrial fibrillation treated with anticoagulation
Source: PLoS One. 2024 Nov 14;19(11):e0312294. doi: 10.1371/journal.pone.0312294 (PMC11563370; doi:10.1371/journal.pone.0312294)

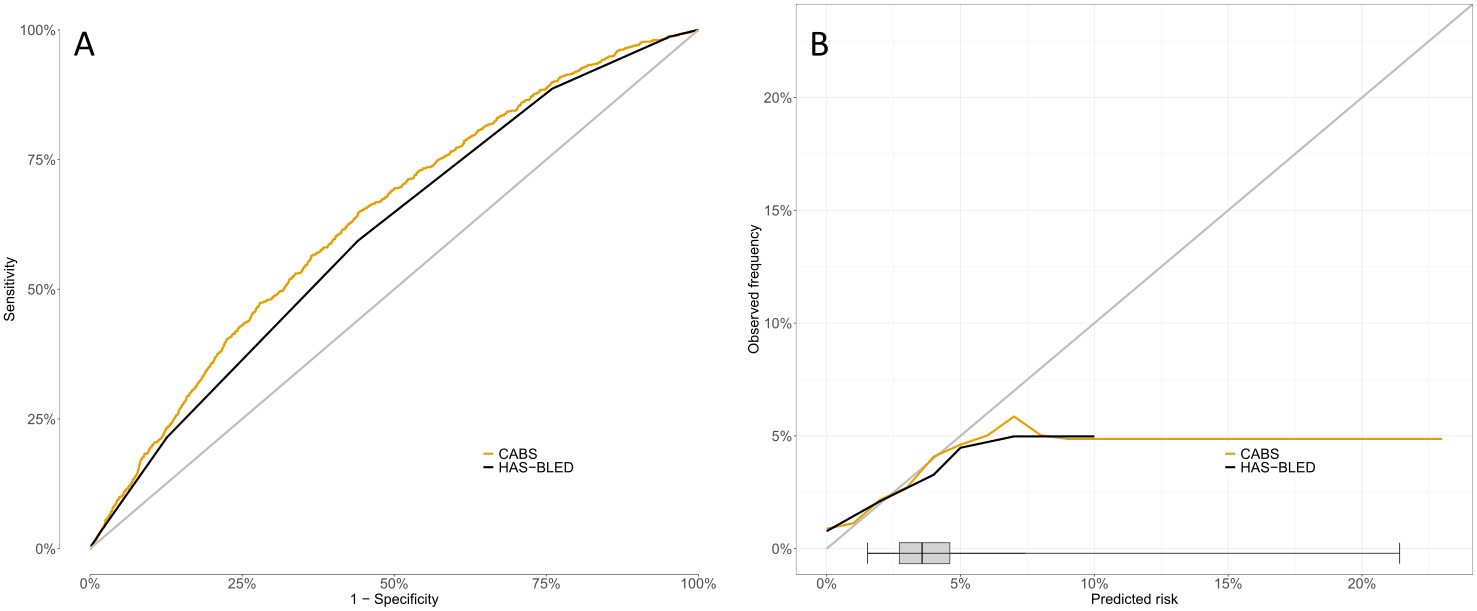

Supplement: S1 Fig — A: Discrimination of the CABS model vs the HAS-BLED score, B: The calibration of the CABS model vs the HAS-BLED across bleeding risk by predicted risk increments. Along the x-axis is shown the distribution of predicted risks. (TIF) [file pone.0312294.s005.tif]

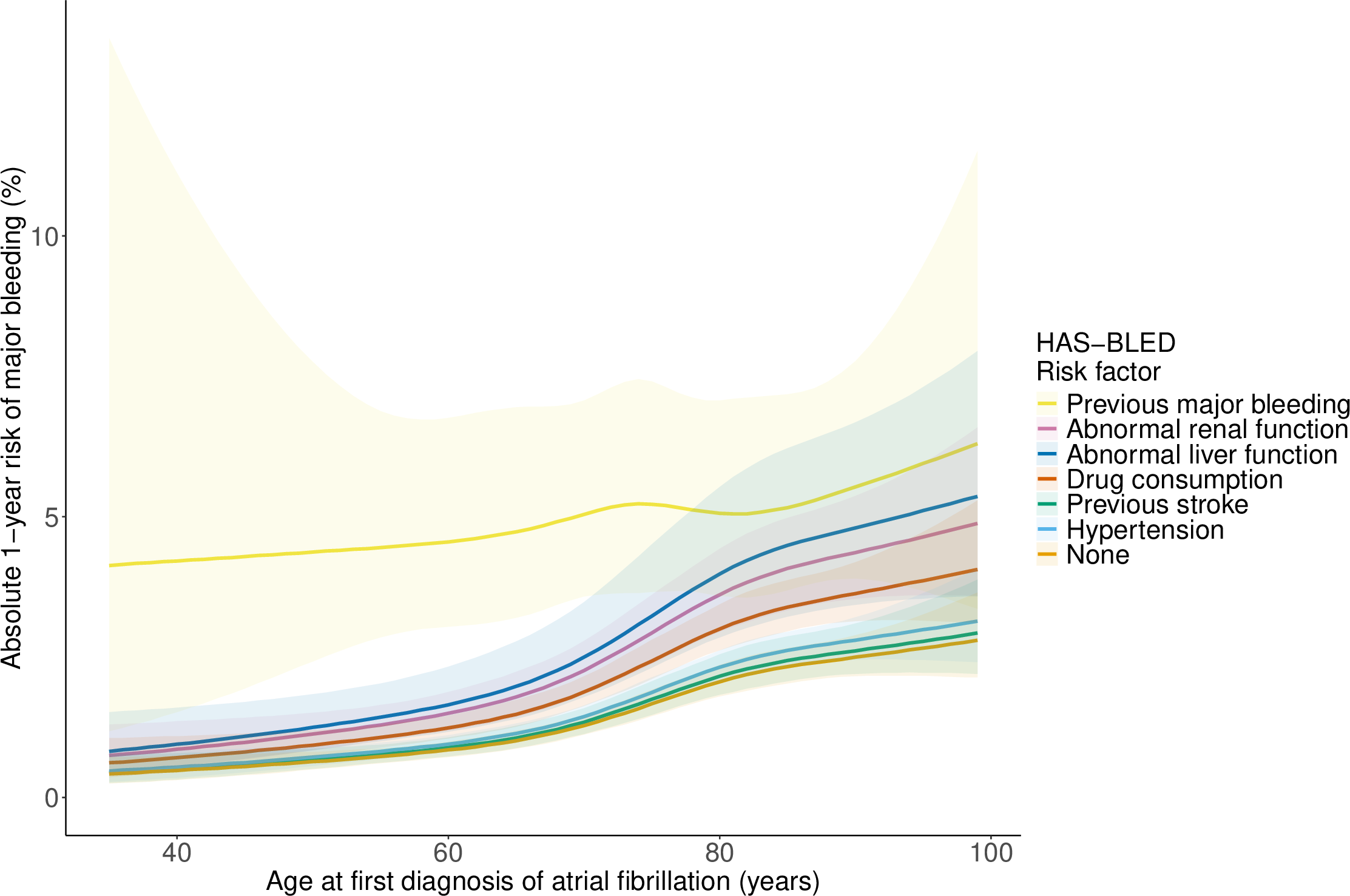

Supplement: S2 Fig — (TIF) [file pone.0312294.s006.tif]

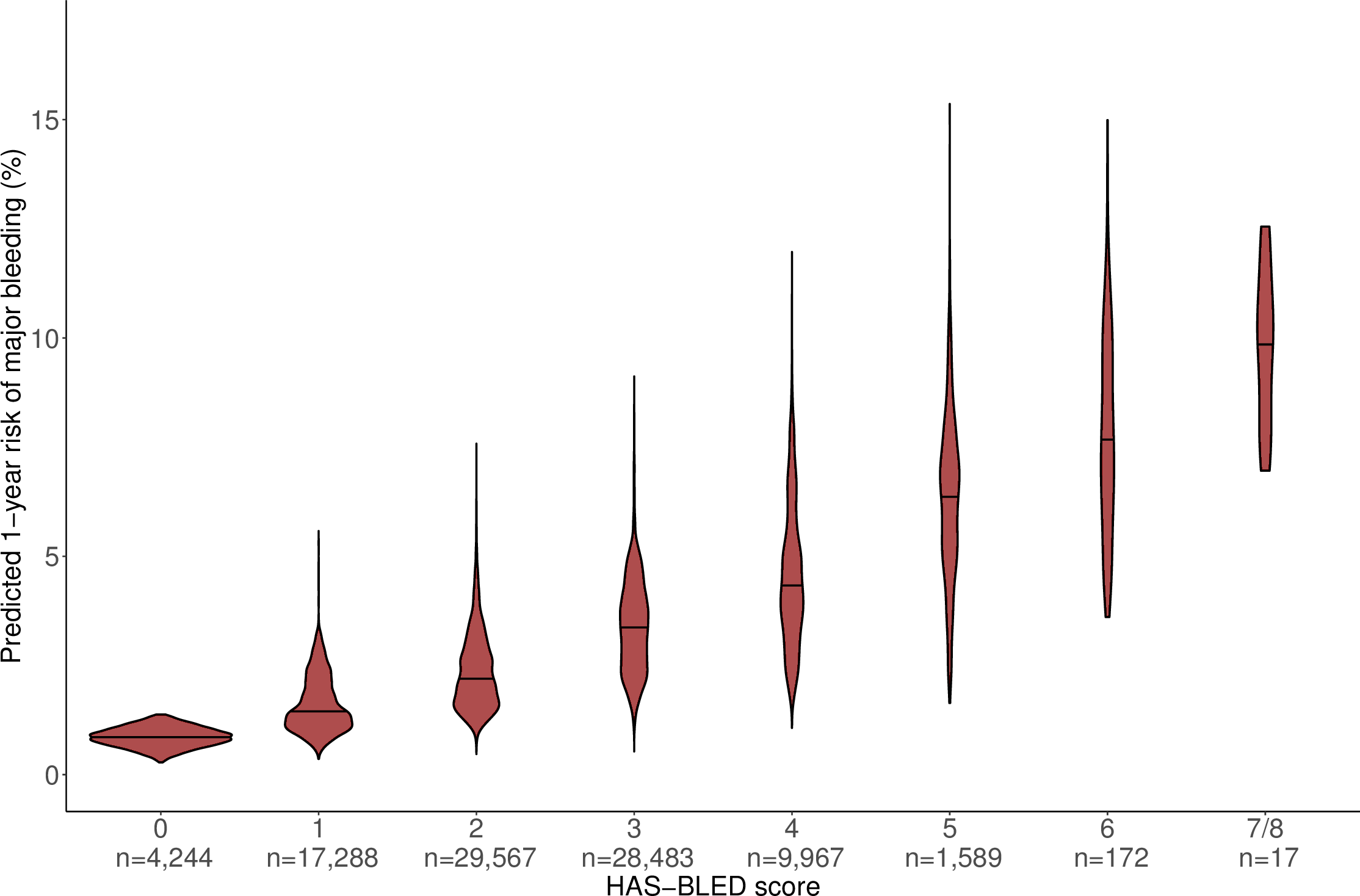

Supplement: S3 Fig — The column width shows the within-column distribution of predicted risk and the number of patients assigned a given HAS-BLED score is indicated at the x-axis. (TIF) [file pone.0312294.s007.tif]
